# Finite Element Analysis of Rubberized Concrete Interlocking Masonry under Vertical Loading

**DOI:** 10.3390/ma15082858

**Published:** 2022-04-13

**Authors:** Amin Al-Fakih, Mohammed A. Al-Osta

**Affiliations:** 1 Interdisciplinary Research Center for Construction and Building Materials, King Fahd University of Petroleum and Minerals, Dhahran 31261, Saudi Arabia; malosta@kfupm.edu.sa; 2 Department of Civil and Environmental Engineering, King Fahd University of Petroleum and Minerals, Dhahran 31261, Saudi Arabia

**Keywords:** crumb rubber, interlocking brick, rubberized concrete, finite element, ANSYS

## Abstract

Fine aggregate and cement have been partially replaced by 10% and 56% crumb rubber and class F-fly ash, respectively, in order to manufacture rubberized concrete interlocking bricks (RCIBs). The newly developed product has been used for masonry construction without the need for mortar (mortarless), and the experimental testing under compression load was investigated by Al-Fakih et al. Therefore, in line with that, this study carried out finite element (FE) analysis for experimental result validation of masonry walls and prisms made of RCIBs. ANSYS software was utilized to implement the FE analysis, and a plasticity detailed micro-modeling approach was adopted. Parametric studies were carried out on masonry prisms to investigate the effect of the slenderness ratio and the elastic modulus of grout on the prism behavior. The results found that the adopted FE model has the ability to predict the structural response, such as compressive strength, stiffness, and failure mechanism, of the interlocking masonry prisms with about a 90% agreement with the experimental results. Based on the parametric studies, the compressive strength for a 6-course prism is approximately 68% less than a 3-course prism and 60% less than a 5-course prism, which means that the slenderness ratio plays a vital role in the behavior of the RCIB masonry prism under the vertical compression load. Moreover, the results showed that the difference between FE and experimental results of the walls was less than 16%, indicating a good match. The findings also reported that masonry walls and prisms experienced higher ductility measured by the post-failure loading under compression. The finite element model can be used for further investigation of masonry systems built with rubberized concrete interlocking bricks.

## 1. Introduction

Masonry, whether traditional or dry-stacked, has a heterogeneous and discontinuous nature and nonlinear material behavior. This is partly attributed to the interfaces between its elements, including the masonry block assembly, the mixture of filler material (mortar), and grout. For mortarless (dry-stacked) masonry, the interface behavior at the dry bed and head joints are much more complicated due to the inevitable air space in the uncontacted region (bed or head joints). The stress field is even more complicated throughout the surrounding continuum [1,2,3].

Nowadays, computer simulation studies have become an easy and appropriate method to determine the structural behavior of many civil engineering structures, including concrete, steel, and masonry. Some of the simulation methods include the extended finite element method [4], damage mechanics [5], discrete element methods [6,7,8], and finite element methods [9,10]. Nonlinear finite element analysis technologies are the most powerful and readily available for masonry subjected to quasi-static monotonous loading and are some of the most advanced structural analysis approaches that take into account various sources of nonlinearity such as material and geometry [9,10]. Although the interlocking mortarless masonry system is not new, few reported masonry studies using finite element analysis have been found, especially focused on this problem. The following numerical studies were reported, which depend mainly on the type of blocks used to assemble the walls. Three finite element modeling methodologies for the investigation of masonry structures were used to simulate the masonry complexity, namely detailed and simplified micro- and macro-modeling. In the detailed micro-modeling method, bricks and mortar are presented by continuum elements, while the interface between the mortar and unit is presented by discontinuous elements. The simplified micro-model is the same as the detailed micro-model, but the joints and interface elements are re-defined as individual elements to represent a contact area. In contrast, in the macro-modeling approaches, the distinction between individual units and joints is not made [11,12,13,14,15].

Senthivel and Lourenço [16] carried out a nonlinear finite element analysis to model the behavior of stone dry-stacked masonry shear walls under combined loading (axial and lateral). The micro-modeling approach and multi-surface interface model are followed in this analysis, where bricks and joints are assumed elastic and inelastic, respectively. The results showed a good agreement between the finite element and laboratory test results. Martínez and Atamturktur [17,18] validated the experimental results of reinforced dry-stacked concrete masonry walls subjected to out-of-plane loading using finite element models. A perfectly plastic model using the Willam–Warnke criterion is implemented. Concrete masonry units and grout are modeled using the SOLID65 element from the ANSYS 15.0 library, while brick-to-brick contact is modeled using surface-to-surface contact elements (CONTA174 and TARGE170 elements). The results showed that an increase in the unit and grout compressive strength enhances the ultimate lateral load-carrying capacity as well as the ductility of the dry-stacked wall. In general, the numerical and experimental results corresponded well. Oh [19] developed a finite element model to simulate the behavior of interlocking mortarless blocks established at Drexel University. The method established to simulate the contact behavior of the dry-stack joint, including geometric imperfection of the dry joint, is adequate, mostly for the modeling of small masonry assemblies. Material nonlinearity is not known to compensate for masonry behavior close to the ultimate load and to anticipate the failure mode. In order to model the joint and block as a homogenous object, Alpa et al. [20] proposed a macro model that follows the homogenization methods. This model focuses on the moving structure of the dry joint, which includes a precise joint. Without adjustment, it is impossible to use the model for structural implementation because it lacks important problems like gradual failure of material, nonlinearity of material, and joint imperfections.

Thanoon et al. [21,22] developed an incremental-iterative program that used a plane stress 2D continuum finite element approach to anticipate the performance and failure mechanism of an interlocking mortarless system under compression. For interface failure, the Mohr-Coulomb criterion was used, and the behavior of interlocking prisms was predicted. The experimental and finite element results were found to be in good agreement. Shi et al. [23] simulated the interlocking brick prisms using a detailed 3D FE model in ABAQUS, where the results of the FE modeling were compared to the results of the experimental findings. The damage and failure patterns of interlocking brick prisms were shown in both laboratory experiments and numerical simulations. Oliveira et al. [24] concluded that the concrete damaged plasticity model, using a micro-modelling approach, was demonstrated to be effective for simulating the behavior of dry-stacked walls at ambient and high temperatures. Chewe Ngapeya and Waldmann [25] carried out an experimental and analytical analysis of the load-bearing capacity of dry-stacked masonry wallets and concluded that, with a mean accuracy of 93%, the suggested mathematical model could predict the loadbearing capacity of dry-stacked masonry.

In addition to the experimental work reported by Ayed et al. [26], and in order to illustrate the effect of local stress on the compressive behavior of masonry, a finite element technique has also been performed. The ABAQUS module interaction is used to model the surface-to-surface contact behavior between the interlocking bricks. The experimental yield pseudo linear load of compressed interlocking blocks is slightly underestimated by the linear elastic model with yield tensile stress, according to the findings.

In line with the experimental work by Andreev et al. [27], the effect of joint geometry and material stiffness on the process of joining dry joints in refractory ceramic masonry was studied utilizing FEM analysis. The authors used ANSYS software to implement the finite element analysis. The elastic-plastic model was adopted, and the results showed that the brick material with elastic-plastic behavior could obtain joint closure curves similar to the experimental results with the flattening curve. Moreover, Ngapeya et al. [28] established a finite element method to examine the influence of the geometric imperfections, including the height variation of different blocks (∆H) and the roughness of the contact area (∆h), on the structural behavior of the dry-stack masonry walls. The authors modeled the dry-stacked masonry block as a 3-D micro-model with consideration of the crushing and cracking capability by using SOLID65 cubic elements provided by ANSYS 17, while the dry joint was modeled as LINK elements, which ensure a load transfer through the actual contact areas. Alternatively, the Coulomb friction law governs the behavior of joints in dry-stacked masonry [29,30].

Recently, a rubberized concrete interlocking brick has been developed [1,31,32,33]. It is categorized as heterogeneous and discontinuous due to the interface between its constituent materials, namely, brick unit, dry joint, and grout. Finite element modeling (FEM) was carried out in this research to simulate the behavior of hollow and grouted rubberized concrete interlocking masonry prism and hollow wall under axial compressive loading until failure in order to validate the experimental results obtained by Al-Fakih et al. [1].

## 2. Materials and Models

Fine aggregates, cement, crumb rubber, fly ash, and water were utilized to make rubberized concrete interlocking brick. Ordinary Portland cement (OPC) has specific gravity and loss of ignition of 3.15% and 2.2%, respectively, confirming the requirements of ASTM C150 [34]. Class F-fly ash has a specific gravity of 2.38, obtained from a coal fire power plant, and has been partially used to replace cement by volume. The wash river sand has a specific gravity of 2.57 and a maximum particle size of 1.2 mm. Mesh #30 (60 microns), nominal size, crumb rubber particles with a specific gravity of 0.95 have been used to replace sand by volume partially. Moreover, the partial replacement of cement and fine aggregate by fly ash and crumb rubber was 56% and 10%, respectively, by volume. A mix ratio of 1:2 (cementitious materials (OPC and FA) to fine aggregates (sand and CR)) was adopted with a water to cement ratio of 0.27. The ingredients and chemical composition of the components employed have already been detailed in Refs. [1,31,32,35].

### 2.1. FE Model

Rubberized concrete interlocking masonry categorizes as heterogeneous and discontinuous due to the interfaces between its constituent materials, namely, brick unit, dry joint, and grout. The finite element (FE) technique is implemented to simulate the behavior of both hollow and grouted rubberized concrete interlocking masonry under axial compressive loading until failure. A detailed micro-modeling approach was followed where each constituent of rubberized concrete interlocking masonry (brick and dry joint) connected to each other by surface contacts at their actual position. A nonlinear 3-D finite element model was developed using the commercial FE modeling package (ANSYS R19.1), and the observed behaviors are validated with the experimental results. Two masonry systems were modeled, including prisms (hollow and grouted) and walls (hollow). Once this stage was accomplished, two parametric studies were carried out, namely: (1) effects of modulus of elasticity of grout on the behavior of grouted masonry prism; and (2) effects of the number of courses (slenderness ratio) on the strength of both hollow and grouted prisms made of RCIB.

### 2.2. Geometry and FE Mesh

**Figure 1:**
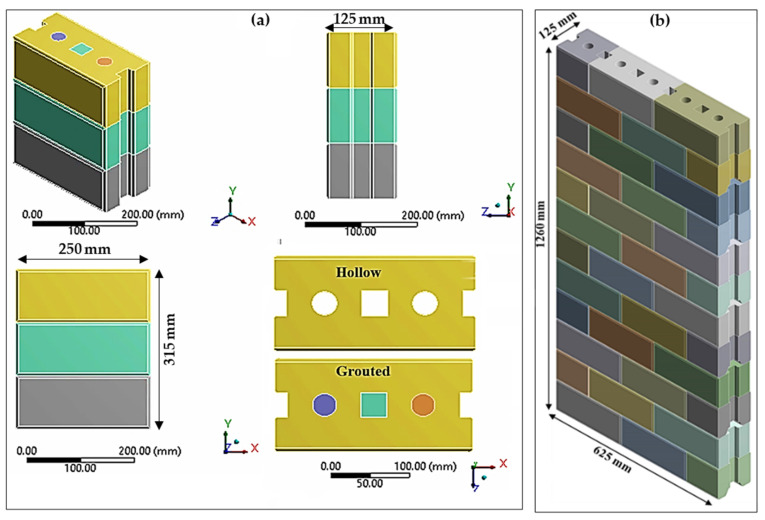
Geometry of RCIB masonry (**a**) hollow and grouted prisms and (**b**) wall.

A compatible 3D FEM geometry of rubberized concrete interlocking masonry prisms and walls was established similar to those tested experimentally. The geometry of the hollow and grouted prisms consists of three courses and two dry joints, as shown in Figure 1a. Alternatively, the size of the hollow wall was chosen to be 625 mm (two and a half units) in length, 1260 mm (twelve courses) high, and 105 mm in thickness, which is similar to the laboratory test specimens as shown in Figure 1b.

**Figure 2:**
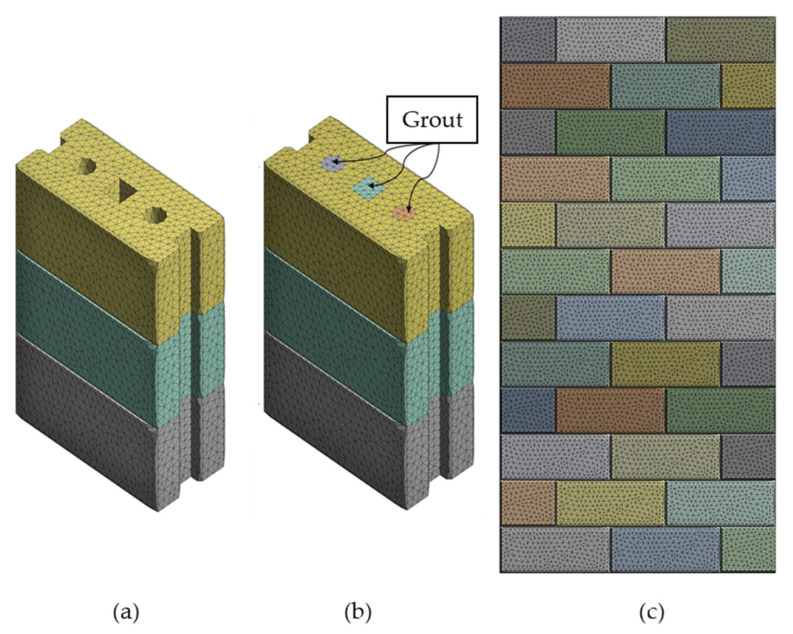
Mesh of the FE model of rubberized concrete interlocking masonry (**a**) hollow prism, (**b**) grouted prism, and (**c**) hollow wall.

**Figure 3:**
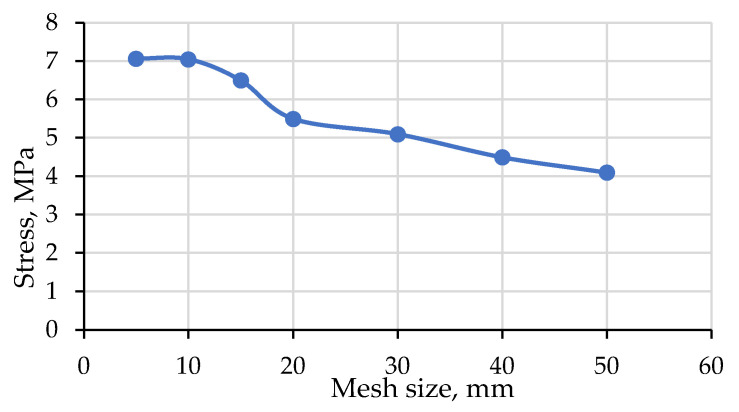
Mesh convergence study on hollow prism.

The meshing convergence analysis was carried out on the hollow prism to study the appropriate mesh size that achieves accurate results. The limit of meshing size/number of elements is considered a converged solution for the FE model once the resultant compressive loading does not significantly change at a certain limit of refinement. The mesh size was gradually reduced from 50 mm to 5 mm. Figure 2 illustrates the convergence study on the relationship between the stress and the number of mesh used for the hollow prism. It can be seen that the mesh size of 10 mm was the best mesh where additional refinement is given the same stress. Therefore, a relatively fine tetrahedral mesh with a 10 mm element size was used for both the hollow and grouted prism as well as the hollow wall (Figure 3).

In this context, the elements chosen to compose the model are the solid finite element form and the contact element type. The solid element of type SOLID187, in ANSYS R19.1, was chosen to represent brick and concrete grout because it can simulate deformations of almost incompressible elastoplastic materials and completely incompressible hyper-elastic materials. SOLID187 is a 3-D tetrahedral higher-order element with ten nodes and three degrees of freedom at each node. For simulating the dry brick-to-brick interface, pair-based contact (surface to surface) elements were used to reflect the discontinuity between units in dry-stack construction. Thus, a contact pair consists of one 3-D contact element with zero thickness called CONTA174 and one 3-D target element with zero thickness called TARGE170. The contact element can support friction in the tangential direction to the contact surface. The friction coefficient (µ) was taken as 0.85 according to the study reported by Oh [19] for dry-stack interlocking bricks. The same contact pair element was also used to contact the grout and brick unit in grouted masonry prisms. The grout in the holes was simulated monolithically together with the prism’s height, and complete bonding between the grout and the RCIBs was assumed.

### 2.3. Constitutive Model

The proposed model is classified as a “detailed micro-model” where the brick was represented by a solid finite element, and the contact interface (dry joints, head, and bed joints) was represented by nonlinear target and contact elements. Target and contact elements were combined to describe the mechanical and structural behavior of a typical joint. The joint model was built using two nonlinear elements, one target and one contact (connected in parallel). All elements were defined according to existing elements in ANSYS R19.1.

Due to the isotropic behavior and elastoplastic response of rubberized concrete interlocking bricks, a nonlinear plasticity model was selected. Therefore, multilinear stress-strain constitutive law was employed in this study. The von Mises yield criterion is combined with the hardening parameter in the multilinear isotropic hardening (MISO). According to the von Mises yield criterion, the material is assumed to yield once the equivalent stress ($\sigma_{e}$) is larger than the current yield stress ($\sigma_{y}$). This can be expressed as Equation (1). On the other hand, von Mises or equivalent strain $\varepsilon_{e}$ is computed by Equation (4).

(1) $$\sigma_{e}>\sigma_{y}>\sqrt{3J_{2}}$$

where $J_{2}$ is the second invariant of the deviatoric stress.

The equivalent stress (von Mises stress) can be expressed as Equations (2) and (4):

(2) $$\sigma_{e}=\sqrt{\frac{1}{2}{\left(\sigma_{1}-\sigma_{2}\right)}^{2}+{\left(\sigma_{2}-\sigma_{3}\right)}^{2}+{\left(\sigma_{3}-\sigma_{1}\right)}^{2}}$$

(3) $$\sigma_{e}=\sqrt{\frac{3}{2}}s_{ij}s_{ij}$$

where $\sigma_{1},\sigma_{2},\sigma_{3}$ are principal stresses, $s_{ij}$ is deviatoric stress.

(4) $$\varepsilon_{e}=\frac{1}{\left(1+\nu^{\prime }\right)}\sqrt{\frac{1}{2}{\left(\varepsilon_{1}-\varepsilon_{2}\right)}^{2}+{\left(\varepsilon_{2}-\varepsilon_{3}\right)}^{2}+{\left(\varepsilon_{3}-\varepsilon_{1}\right)}^{2}}$$

where $\varepsilon_{1},\varepsilon_{2},\varepsilon_{3}$ are principal strains and $\nu^{\prime }$ is effective material Poisson’s ratio.

**Figure 4:**
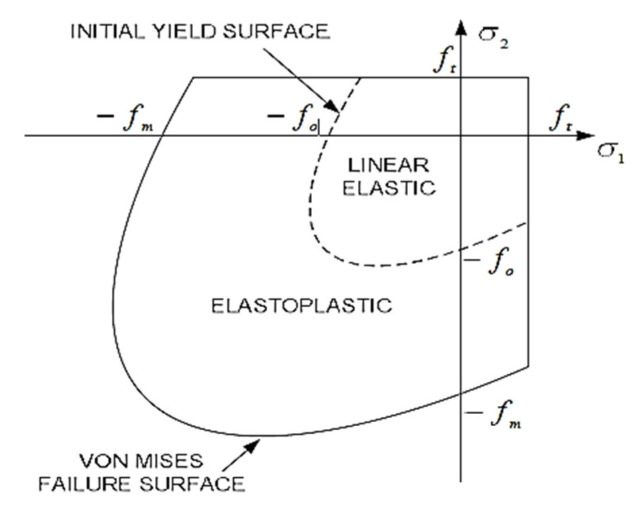
Yield and failure surface [36].

By considering material nonlinearities, the compressive failure and tensile fracture of masonry are governed by a von Mises failure surface with tension cutoff, as shown in Figure 4. in which $\sigma_{1}$ and $\sigma_{2}$ are the principal stresses, $f_{m}$ and $f_{t}$ are compressive and tensile strength of masonry, and $f_{o}$ determines the initial yield surface, which is also governed by the von Mises criterion [36], where assumed in this research.

Before the tension cutoff surface is reached, the material is assumed to be elastic-plastic, of which the plastic behavior is represented by $J_{2}$ plasticity as soon as the stress state reaches the initial yield surface. The material exhibits a strain-hardening behavior when the stress state is between the initial yield surface and the final failure surface. Strain softening occurs once the final yield surface is reached. The von Mises failure criterion can be expressed as follows (Equation (5)).

(5) $$J_{2}-\sigma_{e}^{2}\left(\varepsilon_{e}\right)=0$$

**Figure 5:**
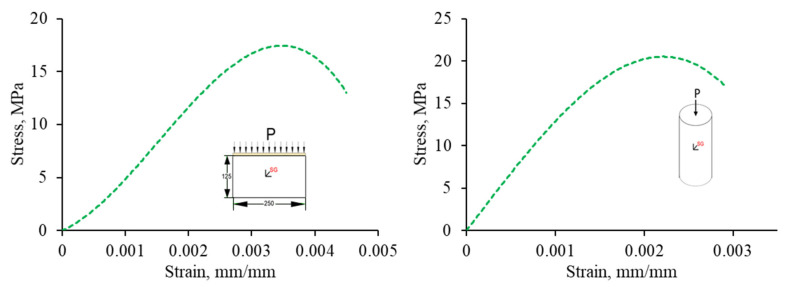
Multilinear stress-strain curve of RCIB (**left**) and concrete grout (**right**).

In this study, the following lists the elastic properties of rubberized concrete interlocking bricks (obtained experimentally) used for the multilinear plasticity model are density (ρ) of 1890 (kg/m^3^), modulus of elasticity (E) of 7416 MPa, Poisson’s ratio (ν) of 0.3, and splitting tensile strength ($f_{splt}$) of 1.15 MPa. Moreover, for concrete grout, the following lists the elastic properties that were used for the multilinear plasticity model are the density (ρ) of 2400 kg/m^3^, modulus of elasticity (E) of 15460 MPa, Poisson’s ratio (ν) of 0.2, and splitting tensile strength ($f_{splt}$) of 3.8 MPa. The determined experimental stresses and strains for both RCIB and concrete grout were used in the model, as shown in Figure 5.

### 2.4. Loading and Boundary Conditions

**Figure 6:**
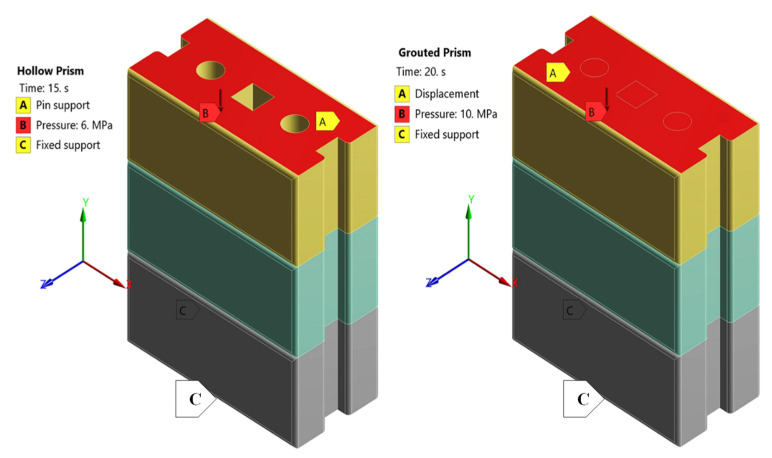
Load and boundary conditions of FE hollow and grouted prism.

Both the hollow and grouted prism models are composed of three courses and two dry joints where the bottom face of the first course is assigned as fix-support while the upper face of the last course receives the loading and is assigned to be a pin-support (no side sway). Figure 6 shows the direction of loading pressure indicated by the downward arrows (B) and the boundary conditions of the model (fixed (C) and pin (A)). Large deflection and nonlinear effects were involved in the finite element analysis with multiple sequential load steps. A total compressive pressure of 10 MPa for the grouted prism and 6 MPa for the hollow prism in the negative Y-direction was applied monotonically at an increment of 0.5 MPa up to the maximum compression load. Generally, each loading increment was applied in a minimum of five iterations (five load substeps), with up to a maximum of 20 equilibrium iterations being used for each substep. The failure was checked throughout the analysis iteration at each brick for compression and the contact interface for tensile and shear based on the aforementioned failure criteria. A complete Newton-Raphson iterative technique and a sparse direct equation solver described in ANSYS 19.1 were used in the solution control. Converged solutions using a 0.5% force convergence value were acquired for all simulated models.

**Figure 7:**
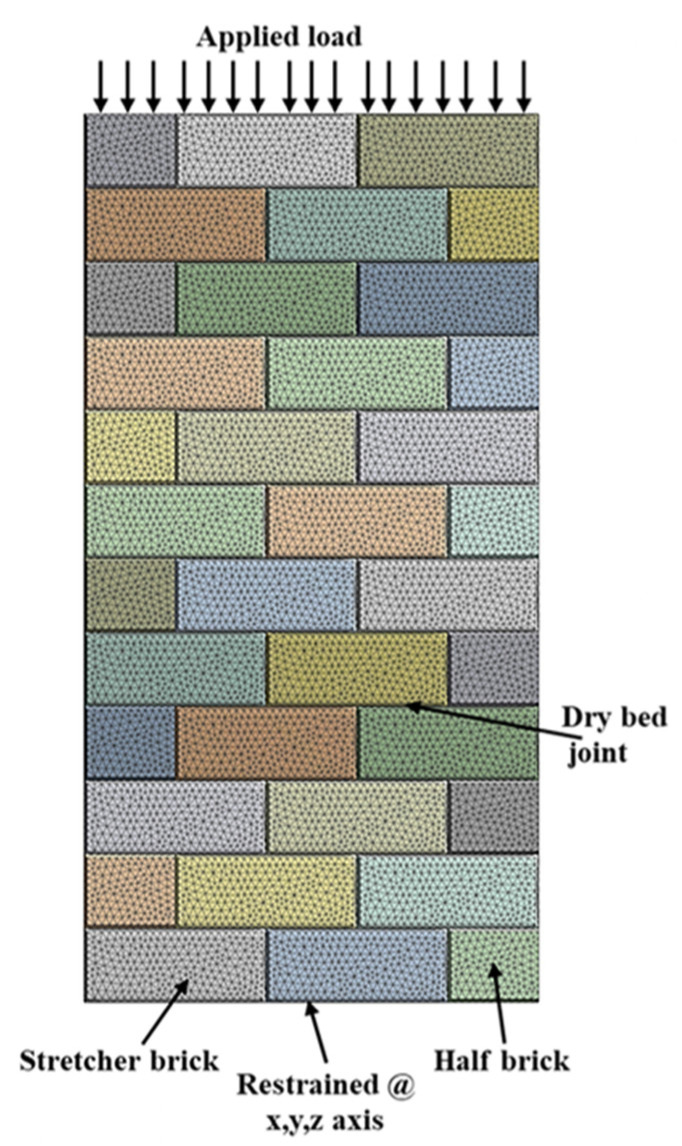
Load and boundary conditions of FE hollow wall.

The wall panels have been restrained at the bottom in three directions (x=0,y=0,z=0) and assigned a displacement (x=0,y=free,z=0) at the top face shell of the model, as shown in Figure 7. Iterative FEM analyses with actual test boundary conditions were carried out until the FEM result reasonably matched the physical test results. The loading of the model was applied by means of uniform pressure (σ = 5 MPa) for a hollow wall over the top surface of the wall, as shown in Figure 7. Because of nonlinearity, slight increases in load (0.5 MPa) have been applied to allow convergence of the solution. Convergence is accomplished if, at all integration points in the structure, the plasticity ratio (plastic strain ($\varepsilon_{pl}$)/ elastic strain ($\varepsilon_{el}$)) is less than a present value.

## 3. Results

### 3.1. RCIB Masonry Prisms (Hollow and Grouted)

The comparison between the experimental and finite element results of both hollow and grouted rubberized concrete interlocking prism is shown in Table 1, where $k_{exp}$, $P_{exp}$, and $E_{exp}$ are the stiffness, ultimate compression load, and modulus of elasticity, respectively, attained experimentally, whereas $k_{FE}$, $P_{FE}$, and $E_{FE}$ are the corresponding results attained from finite element analysis.

**Figure 8:**
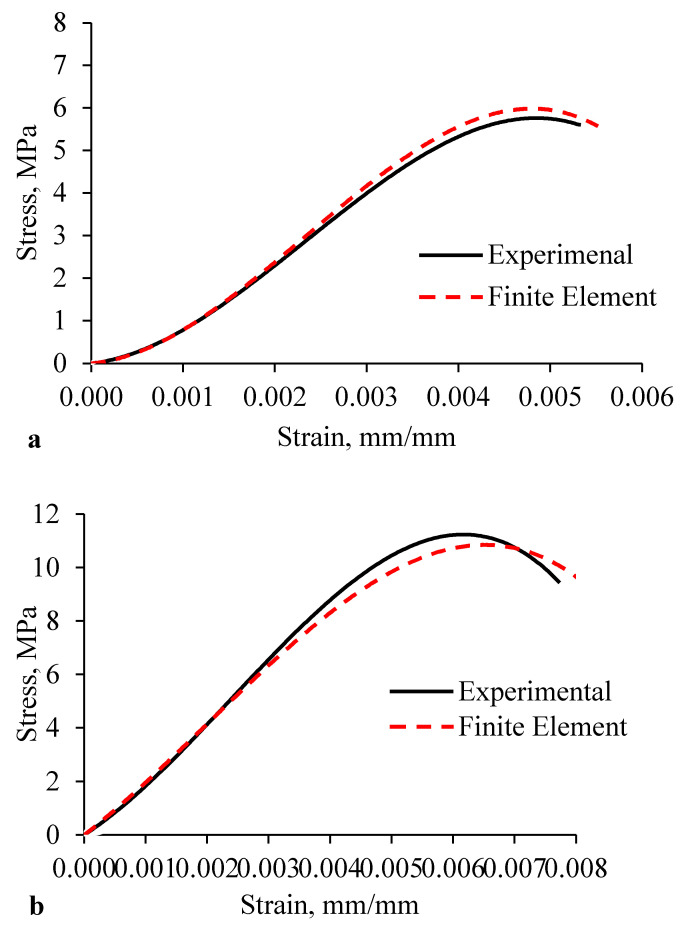
Comparison of the FE and experimental axial stress-strain curve. (**a**) Hollow prisms and (**b**) grouted prisms.

Stress-strain relations calculated by the FEM models are compared to those from corresponding test curves, as shown in Figure 8. Experimentally, the ultimate compressive stress was calculated by dividing the ultimate applied compression load by the bearing area (loaded area). The strain was obtained from the reading of the LVDTs attached to the prism, and then the final stress-strain curves were plotted.

The ultimate loads obtained from the hollow and grouted FEM model were 132 and 304 kN, respectively, which was 16.9% higher than and 0.31% lower than the experimental results of the hollow and grouted prism, respectively. The high estimation of the peak compressive load of the hollow prism by the FE model was caused by elastic-plastic behavior with hardening used in the material model and other possible defects in the hollow prism. In general, a good agreement was demonstrated for all comparisons, confirming the reliability of the finite element idealization.

The experimental modulus of elasticity of the rubberized concrete interlocking hollow prism of 4735 MPa was increased to 5284 MPa while reduced from 7654 MPa experimentally to 7441 MPa on FE for grouted prism. These differences are due to the complex combined effect of the material and dry contact nonlinearity. However, the elastic modulus predicted by the finite element model compares very well with that attained from the experimental testing (ΔE≤10), as shown in Table 1.

**Figure 9:**
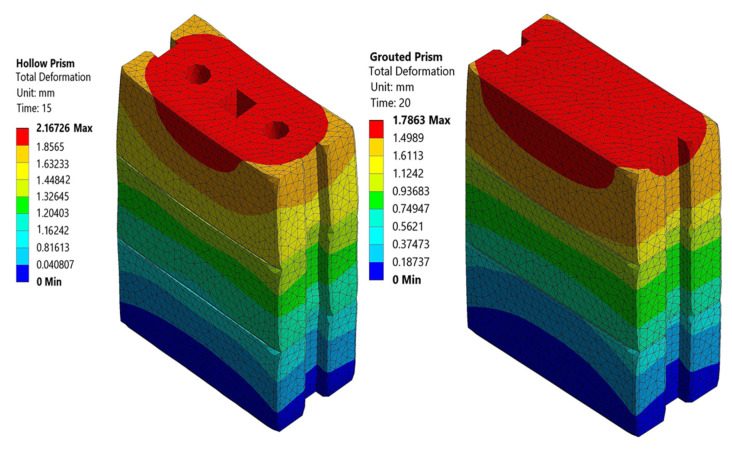
Deformation of hollow and grouted FE model of the rubberized concrete interlocking prism.

The total average deformation of hollow and grouted specimens was measured experimentally by LVDTs to be 2.55 mm and 2.16 mm, respectively. The simulation output predicted the deformation to be 2.167 mm and 1.786 mm for the hollow and grouted FE models, respectively (Figure 9). It can be seen that the test of FE ratio of axial deformation was close to unity (1.17 and 1.2), which implies 83% and 80% agreement.

**Figure 10:**
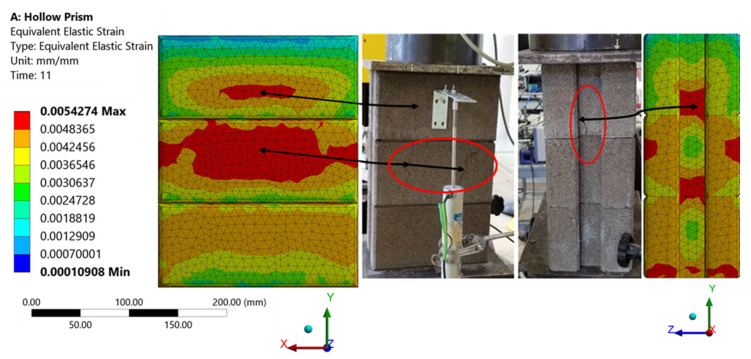
Failure mode (equivalent elastic strain) of hollow prism experimentally and FE model.

**Figure 11:**
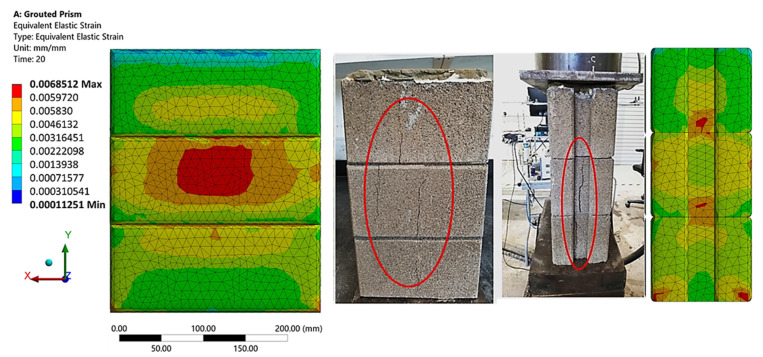
Failure mode (equivalent elastic strain) of grouted prism experimentally and FE model.

The strain redistribution due to the yielding of the rubberized concrete interlocking brick indicates the failure mechanism up to the ultimate failure of the prism. Figure 10 and Figure 11 show a typical failure mode of the hollow and grouted FEM model under compressive loading from the Y-direction. With the progressive increase in the compressive loading and contact stiffness, the triaxial state of stress develops in two regions: (1) near the dry joint where the gap is opened and (2) the middle of the web and the face shell where longitudinal compressive stress is intense. The first type of tensile stress is directly induced by the lateral expansion at the opened gap and the presence of grout, while the latter is induced by the Poisson’s effect (especially in the presence of crumb rubber that has a higher Poisson’s ratio). As a result, a good portion of the face shell and webs would be subjected to high compressive stress. These cracks are highlighted in circles and shown in Figure 10 for the hollow prism and Figure 11 for the grouted prism. Thus, the resulting failure modes were observed to be shear compression failure and web splitting. These confirmed the failure mechanism observed on the laboratory test. Despite the fact that the cracking did not occur at the same places as in the experiments, the cracking pattern was properly anticipated.

Generally, for interlocking masonry hollow or grouted prisms, the average experimental to FE ratios of ultimate compression load, peak stress and strain, stiffness, and modulus of elasticity were all close to unity. As a result, it was confirmed that the FEM model produces stiffness and strength values that correlate well with laboratory test findings; thus, it gives a valuable platform for simulating and understanding the behavior and failure mechanism of the rubberized concrete interlocking masonry prism.

### 3.2. RCIB Masonry Wall (Hollow)

The established FEM models of the rubberized concrete interlocking masonry wall panels were validated with the laboratory test results discussed in [1]. The structural properties obtained from the FE simulation were tabulated and used as a means of comparison of the laboratory test results, as shown in Table 2.

Table 2 shows the comparison between the experimental and finite element results of both hollow and grouted rubberized concrete interlocking wall panels. The experimental compressive strength of the rubberized concrete interlocking hollow wall panel of 3.87 MPa was slightly decreased to 3.73 MPa while highly increased from 5.75 MPa experimentally to 7.62 MPa in the FE model for the grouted wall panel. These differences are due to the complex combined effect of the material and dry contact nonlinearity. However, elastic modulus, stiffness, and axial deformation predicted by the finite element model compared well with that attained from the physical testing.

**Figure 12:**
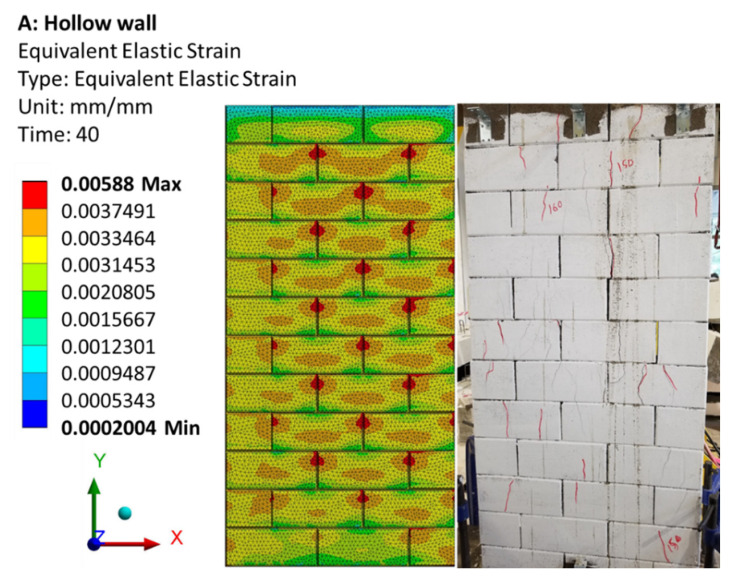
Failure mode (equivalent elastic strain) of hollow wall experimentally and FE model.

The FE failure mechanism is presented as the equivalent elastic strain for both hollow and grouted wall panels and then compared with the failure mode of the tested specimens, as shown in Figure 12. It is clear that the failure mode and strain concentration are comparable in both FE and experimental walls. With the progressive increase in the compressive loading and contact stiffness, the triaxial state of stress develops in two regions: (1) near the dry joint where the gap is opened and (2) the middle of the web and the face-shell where longitudinal compressive stress is intense. These confirmed the failure mechanism observed on the laboratory test and described in Section 4.3 of Ref. [1]. Even though the cracking was not at the exact locations as those in the experiments, the failure mode was accurately predicted. In general, it was observed that two major failures were exhibited for both hollow and grouted wall panels—a shear mode failure along the bed or head joint and a flexural failure of the wall.

### 3.3. Parametric Study of RCIB Masonry Prism

The possible parameters that can be changed are the height of the specimen (h) and the modulus of elasticity of grout ($E_{g}$). The finite element model and related parameters utilized in this parametric study are tabulated in Table 3. The boundary conditions, constitutive model, loading procedures, and analysis were as previously described in Section 2.1, Section 2.2, Section 2.3 and Section 2.4. The first parametric study explored the effect of the slenderness ratio (λ) (height to thickness ratio (h/t)) on the prism behavior for both hollow and grouted systems. Four of each hollow and grouted FEM model were simulated and analyzed. The effect of the elastic modulus of grout on the prism behavior was the second parametric study to be modeled.

**Figure 13:**
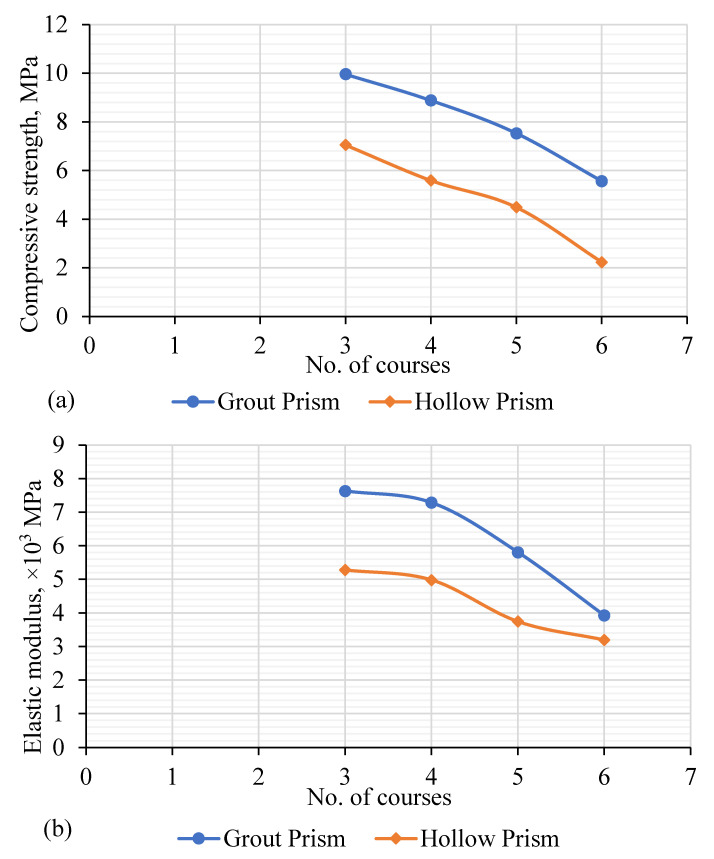
Effects of slenderness ratio on the hollow and grouted prism. (**a**) Prism strength; (**b**) prism elastic modulus.

The effect of the slenderness ratio on the strength of the hollow and grouted FE model of the rubberized concrete interlocking prism is summarized in Table 4. It can be seen that the increase in the number of courses for both hollow and grouted prisms decreased the compressive strength, elastic modulus, and stiffness of prism, either hollow or grouted masonry, as shown in Figure 13. This may be attributed to the fact that the ultimate strength of the masonry system is a function of the slenderness ratio, where the capacity decreases with the increase of the length of the masonry specimen. However, for hollow prisms, a significant effect was detected for six courses. The compressive strength for a six-course prism was approximately 68% less than a three-course prism and 60% less than a five-course prism. For grouted prisms, the presence of the grouted cores provided continuity and thus reduced the slenderness ration effect. The strength of grouted prisms of a six-course prism was 44% less than a three-course prism. For hollow and grouted prisms, the elastic modulus for a six-course prism was 49% and 31%, respectively, of that for the three-course prism.

**Figure 14:**
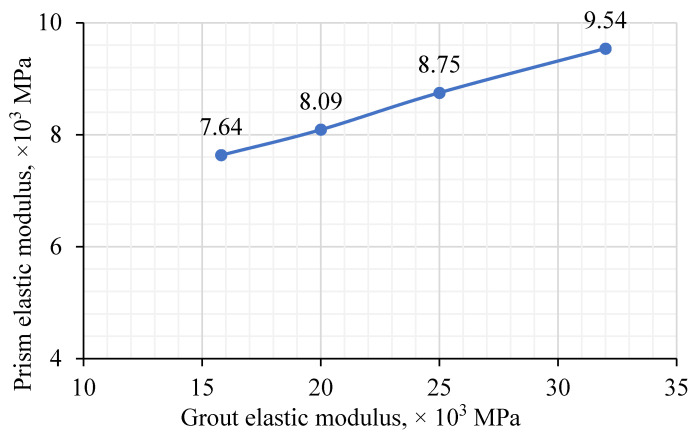
Effect of elastic modulus of grout ($E_{g}$) on elastic modulus of prism ($E_{p}$).

While the modulus of elasticity of the rubberized brick ($E_{b}$) was kept constant at 7416 MPa, the modulus of elasticity of the grout varied from 15,826 MPa to 32,000 MPa. As expected, a linear relation (proportion) was observed. The modulus of elasticity of the prism increased from 7640 MPa to 9540 MPa (Figure 14). This increase implies that an increase of grout elastic modulus greatly affected the prism strength in the rubberized concrete interlocking grouted masonry in two ways: (1) increasing the elastic modulus of grout (strength) leads to an increase in the load-carrying capacity of prisms, and (2) increasing elastic modulus of grout induces higher axial stress in the grout causing the lateral tensile stresses in the brick units to be higher due to the confinement of grout. As a result, the elastic modulus of the rubberized concrete interlocking grouted prism was almost linearly proportional to that of grout, as shown in Figure 14. Increasing the modulus of elasticity of grout by almost 50% led to an average increase in that of the prism by 25%.

## 4. Conclusions

The following conclusion can be drawn:

1. The finite element analysis emphasized the distribution of forces and stresses within the prism and the deformation mechanism of the rubberized concrete interlocking masonry prisms (hollow and grouted); 90% agreed with experimental findings.
2. RCIB walls and prisms experienced higher ductility measured by the post-failure loading under compression.
3. Web splitting and face spalling were the common failure mechanisms for wall panels, while shear compression failure and web splitting were the common failure mode for the hollow and grouted prisms for both the experimental specimens and simulated models.
4. The differences between the FE and experimental results of the walls were less than 16%, indicating a good matching.
5. With an increase in the number of courses, both prism strength and modulus of elasticity decreased.
6. It is recommended to employ another plasticity model such as Willam–Warnke or Drucker–Prager yield criteria instead of the von Misses yield criterion for modeling the rubberized concrete interlocking masonry.

## Figures and Tables

**Table 1:** Experimental and FE results (strength and stiffness).

Prism Type	$k_{exp}(\mathrm{kN}/\mathrm{mm})$	$k_{FE}(\mathrm{kN}/\mathrm{mm})$	Δk(%)	$P_{exp}(\mathrm{kN})$	$P_{FE}(\mathrm{kN})$	ΔP(%)	$E_{exp}\mathrm{MPa}$	$E_{FE}\;\mathrm{MPa}$	ΔE(%)
Hollow	281.19	309.66	9.2	109.65	132	16.9	4735	5284	10.4
Grouted	684.4	618.69	9.6	305.34	304	0.31	7654	7441	2.9

**Table 2:** Experimental and FE findings for tested masonry walls.

Property	Hollow Wall Results			Grouted Wall Results		
	EXP	FE	EXP/FE	EXP	FE	EXP/FE
Ultimate load, kN	181	216	0.84	424.8	510	0.83
Compressive strength, MPa	3.87	3.73	1.04	5.75	7.62	0.75
Elastic modulus, MPa	727.42	845.4	0.86	1778.34	2320.2	0.76
Ultimate strain at failure, mm/mm	0.0055	0.0049	1.12	0.00436	0.00325	1.34
Stiffness, kN/mm	27	35	0.77	103.23	131.2	0.79
Deformation, mm	6.78	5.33	1.27	5.54	4.96	1.12

**Table 3:** Details of FE model for parametric study.

Model		No. of Courses	h (mm)	l (mm)	t (mm)	λ (h/t)	$E_{b}$ (MPa)	$E_{g}$ (MPa)
Grouted	Slenderness effect	3	315	250	125	2.52	7416	15,826
		4	420	250	125	3.36	7416	15,826
		5	525	250	125	4.2	7416	15,826
		6	630	250	125	5.04	7416	15,826
Hollow		3	315	250	125	2.52	7416	15,826
		4	420	250	125	3.36	7416	15,826
		5	525	250	125	4.2	7416	15,826
		6	630	250	125	5.04	7416	15,826
Grouted	Grout effect	3	315	250	125	2.52	7416	15,826
		3	315	250	125	2.52	7416	20,000
		3	315	250	125	2.52	7416	25,000
		3	315	250	125	2.52	7416	32,000
		3	315	250	125	2.52	7416	40,000

**Table 4:** FE results of the hollow and grouted prism with different courses.

Type	No. of Courses	Load *P* kN	Stress, *σ_p_* MPa	Strain, *ε*_p_ mm/mm	Deformation *δ_p_*, mm	Elastic Modulus, *E_p_* N/mm^2^	Stiffness, *k_p_* kN/mm
Grouted prism	3	304.4	9.96	0.00597	1.79	7.64	618.69
	4	277.4	8.88	0.00480	2.17	7.29	542.25
	5	249.0	7.53	0.00289	2.32	5.81	367.82
	6	221.1	5.56	0.00213	5.54	3.93	270.56
Hollow prism	3	132.5	7.05	0.00540	2.167	5.28	309.7
	4	121.7	5.59	0.00656	2.45	4.98	253.3
	5	95.3	4.49	0.00535	2.749	3.75	205.27
	6	75.8	2.23	NA	4.62	3.20	152.15

## Data Availability

Data are available upon request; to those interested, please contact the corresponding author.
